# Description of a novel technique for ultrasound-based planning for gynaecological 3D brachytherapy and comparison between plans of this technique and 2D with fluoroscopy

**DOI:** 10.3332/ecancer.2022.1415

**Published:** 2022-06-23

**Authors:** Juan Carlos Pari Salas, Danny Giancarlo Apaza Véliz

**Affiliations:** 1 Servicio de Radioterapia, Hospital Goyeneche de Arequipa, 04001, Peru; 2 Universidad Católica de Santa María, Arequipa 04013, Peru; 3 Universidad Nacional de San Agustín de Arequipa, 04000, Peru; 4 Departamento de Física, Faculdade de Filosofia, Ciências e Letras de Ribeirão Preto, Universidade de São Paulo, 14040-901, Brasil; a https://orcid.org/0000-0001-5476-0679; b https://orcid.org/0000-0001-7645-2626

**Keywords:** ultrasound, brachytherapy, cervical cancer

## Abstract

With the purpose of implementing a way to obtain volumes from ultrasound axial images, a novel method for 3D gynaecologic brachytherapy was assessed, with a 3D-printed attachment for a commercial stepper (for prostate brachytherapy). It allowed the acquisition of a transabdominal axial image series by ultrasound; these images were uploaded to a treatment planning system where high-risk clinical tumour volume (HR-CTV) and risk organs were contoured. A dose administration plan was developed with orthogonal X-ray images (0° and 270° incidences), using International Commission on Radiation Units and Measurements (ICRU) 38 points. The same plan was applied in the ultrasound images’ sequence and their respective volumes; differences were noted. In the 20 cases analysed, with a given point A dose, its corresponding dose delivered to 90% of HR-CTV percentage was highly variable (mean = 104.2, SD = 26.01). There is a significant variation of this percentage when point A falls outside the HR-CTV than when it falls inside (*p* < 0.00001). There is a significant correlation in terms of the bladder point dose ICRU 38 and the Maximum dose to 2cc of organ or target volume (D2cc) bladder (*p* = 0.021); however, there is no such correlation when we relate the rectum point dose ICRU 38 to the D2cc rectum (*p* = 0.327). There was a negative correlation between HR-CTV and D2cc bladder and D2cc rectum; both were statistically significant. There were significant differences comparing ICRU points and dose to prescription and organ at risk volumes, pointing out that there is room for optimisation of plans using the latter technique. So, it is proposed to further test this image modality and compare it to other imaging techniques that allow the creation of volumes, such as computed tomography or magnetic resonance imaging.

## Background

Brachytherapy for cervical cancer plays a major role in its treatment; its omission leads to decreased survival and local control. Brachytherapy guided by computed tomography (CT) and/or magnetic resonance imaging (MRI) has advantages: visualisation and definition of the volumes, and better tissue discrimination. The available literature suggests an advantage in both local control and survival when using image-guided brachytherapy (IGBT) instead of fixed points according to International Commission on Radiation Units and Measurements (ICRU) 38 [1, 2]. In one study, a trend towards better overall survival and local control with IGBT was shown [1], while a meta-analysis showed better survival and local control, as well as a lower toxicity profile [2]. According to the published literature, there is good concordance between the measurements of ultrasound images and those of pelvic MRI [3–5]. Compared to CT, it is perceived that ultrasound images are more discriminative and accurate, in the appropriate context. From this viewpoint, we have developed a technique of ultrasound images acquisition that, by using a stepper for prostatic brachytherapy with a 3D printed attachment, can obtain axial images with a fixed distance and thus achieve a volumetric reconstruction with these images that allows assessing and defining the high-risk clinical tumour volume (HR-CTV) and the organs at risk. The technique for obtaining these images is described, as well as its incorporation into the planning based on orthogonal radiographic images. We assessed for differences between the doses with 2D conventional planning, adding the ultrasound-based volumes, to see if there is a statistical significance that would warrant more research on the subject.

## Materials and methods

In this study, an attachment was built, which allowed taking ultrasound images in axial orientation, with 5-mm spacing, reconstructed in a brachytherapy planning software for volumetric contouring. Acquisitions of orthogonal X-ray (0° and 270° incidences) and ultrasound images were made, the latter with the attachment already described and treatment planning was carried out with the former, according to the ICRU report 38, administering this treatment to the patient. After the treatment, information on the shape, applicator’s number/position and prescription points/risk organs were recorded, and this information was used to develop a new plan with sequential ultrasound images. An overlap was made, with emphasis on the image of the tandem matching the respective template and bladder point at its corresponding ultrasound images, proceeding to outline HR-CTV and organ at risk, to then analyse the administered plan with the presence of volumes.

### Making of the attachment

An attachment to the stepper was built in 3D printing with polylactic acid, consisting of two decagonal arms bonded by an adjustable clamp. It had one end of the arm placed where the endorectal transducer would be located, secured in the same way, parallel to the patient, while the other arm was located perpendicular to the first. The latter allowed the attachment at its end with a clamp of the convex transducer of 3.5–5 MHz, from a TOSHIBA ultrasound scanner model SSA-4000 (TOSHIBA Corporation, Tokyo, Japan), so that axial images can be sequenced according to a predetermined distance that is controlled from the stepper.

In the case of our technique, images were captured with a laptop using an EASYCAP video capture card and the built-in image capture programme in Windows 8 (Microsoft Corporation, Redmond, Washington, U.S.). After that, a folder was created with the set of images obtained. This folder will become the specific series of images for the patient. We used those images on the high does rate (HDR) plus 3 brachytherapy treatment planning system (Eckert & Ziegler Strahlen- und Medizintechnik AG, Berlin, Germany) for volume contouring, and size calibration of the images, being those in .JPG format, using the scale on the ultrasound images.

### Patient selection

As a requirement, patients who would undergo this imaging technique should not have a conformation with excavated abdomen or a very redundant abdomen. Patients receiving vaginal irradiation were not considered, since it is not possible to have information about this anatomical area with the technique used. All the patients had an anteverted uterus, lateralised to the left in 10 patients and central in 2 patients.

### Patient preparation

In order to have an acoustic window, 180 cc of saline serum at room temperature was placed through the Foley catheter, and a radiopaque rectal catheter was placed. This catheter was placed for the acquisition of images and removed prior to treatment.

### Image acquisition process

**Figure 1.**
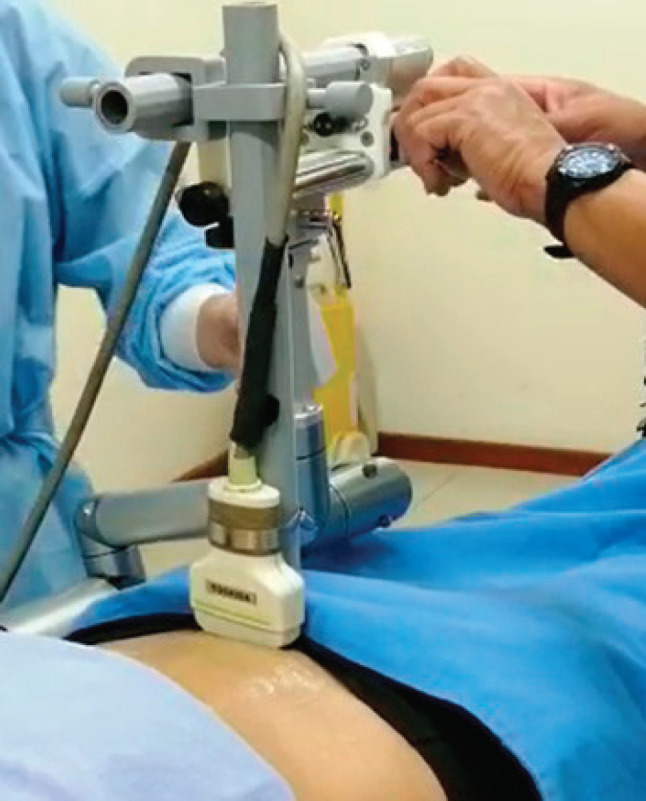
Imaging procedure showing the stepper, the attachment and the transducer in place.

The patient must be in the brachytherapy application room, in the position of application of the treatment, with the applicators already on; previously, fluoroscopic orthogonal images, posterior-anterior and lateral from the right, must have been obtained according to the standard technique. We then proceeded to take a sagittal ultrasound image, in which the tandem, the balloon of the Foley catheter, and the probe placed in the rectum are seen, if possible. After that, the stepper and the attachment are assembled and the transducer is placed on the skin, at the level of the upper aspect of the symphysis of the pubis, as shown in Figure 1. The pressure must be enough to allow coupling with the skin, without distorting it significantly.

The image steps can be 5 and 2.5 mm, with the former preferred because it allows a minor amount of time spent in acquisition, which means less internal movement (i.e., bowel movement and artefacts related to breathing). The acquisition begins in this way, trying to evaluate in each step a correct movement of the transducer, and after that the corresponding digital capture of the image. Two series were made, the first in mode B and the second in mode B with Doppler mode colour activated, taking special consideration in being able to obtain an image of the entrance of the uterine artery at the level of the isthmus, to be able to perform an adequate contouring of the HR-CTV.

### Processing and planning

The images obtained were exported to the HDR plus 3 Treatment Planning System (TPS) and stored for later use. After that, a plan was created with the orthogonal images from the C-arm obtained previously, with the application of the corresponding points according to the ICRU report 38. This conformation, understood as the positioning of the applicator and positioning of the points, together with the application of the plan, in relation to positioning and time of the source in the different steps, was saved for later use.

In the set of sequential images, the magnification was verified, proceeding to make the necessary adjustments, and the position of the applicator in the reconstructed volume was evaluated at first. Subsequently, the following structures were outlined:

**HR-CTV**, as the uterine cervix area and the tumour, for practical purposes will be defined according to the recommendations of Groupe Europeen de Curietherapie- European Society for Therapeutic Radiology and Oncology (GEC ESTRO). The entire volume of the cervix and tumour extending to parametria, which appear as echogenic in both cases, will be contoured up to the isthmus or the entrance of the uterine artery, extending 1 cm towards the fundus direction, in a conical form. The position of the applicator was taken into account to make this projection. The uterine artery can be identified on Doppler examination. For further guidance, the clinical examination previous to the insertion and the CT images at the moment of staging were taken into account.

**Bladder:** In slides where possible, echogenic content and the wall, which is noted as a hyperechogenic interface, are outlined. Slides in which, due to low pressure, there is interposition of gases from the intestinal loops that hinders visualisation were left blank to be completed later by interpolation.

**Rectum:** From two slides prior to the appearance of the HR-CTV, if possible, as far as it can be visible or up to the angle. For the guidance of the contour, we proceeded to follow the path that marks the dummy of the rectal probe and that we evidenced in the image of fluoroscopy. On ultrasound images, we followed the hyperechogenic zone to delimit the rectum properly.

**Figure 2.**
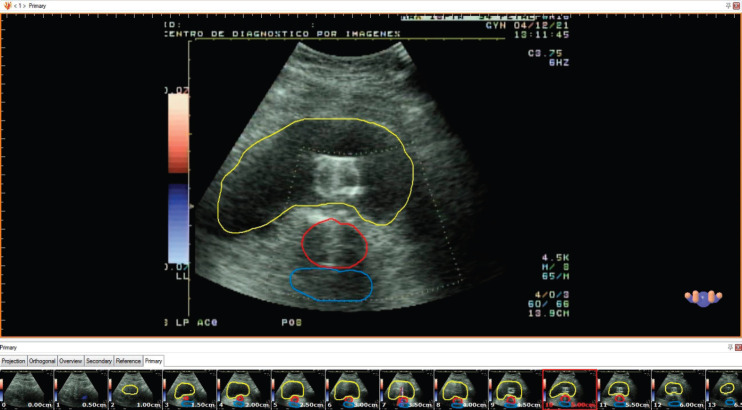
Contouring of the reconstructed images in HDR plus TPS.

An example of this procedure is shown in Figure 2.

### Planning procedure

**Figure 3.**
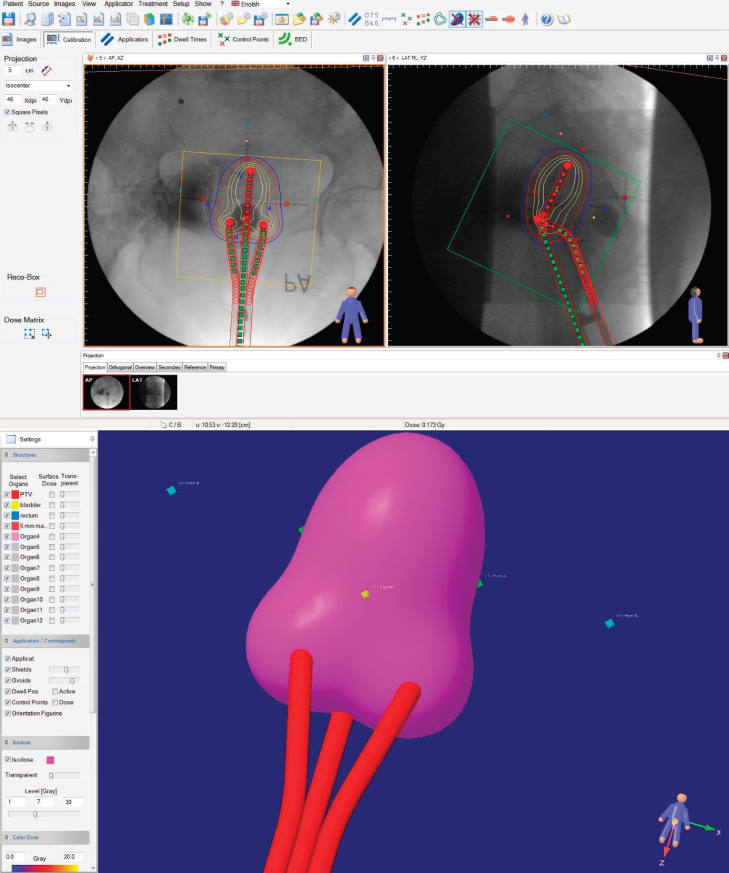
Development of the treatment plan with X-ray images in the HDR plus TPS. The applicators and point positions are saved in a cluster for exporting to the image sequence plan.

The treatment planning was carried out with the C-arm images, placing them in an orthogonal reconstruction, proceeding to place the applicators, as observed in the images, and after that to continue with the creation of the prescription points, as specified in ICRU 38; this is shown in Figure 3.

**Figure 4.**
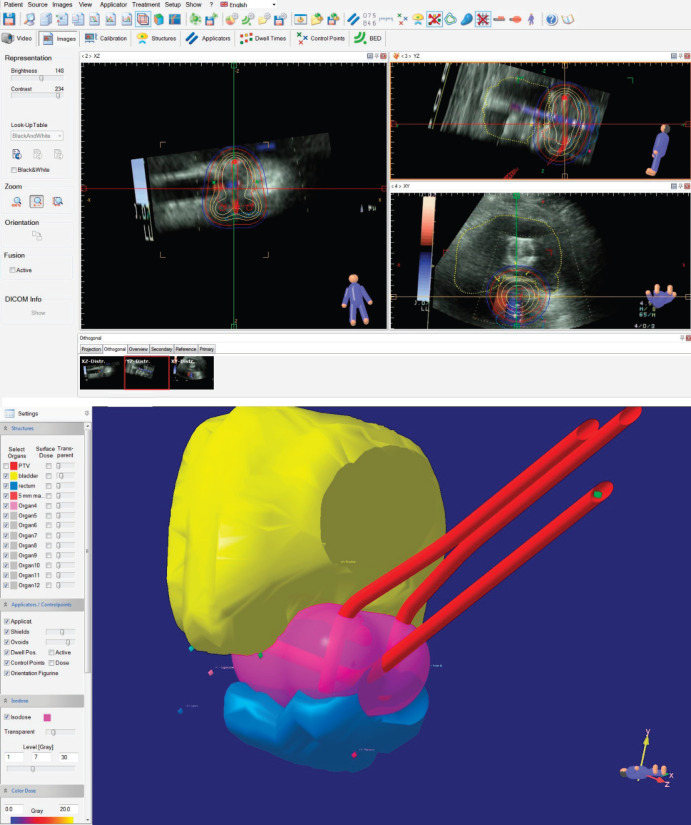
Placement of the ICRU 38 point and applicator cluster in the image sequence reconstruction.

Once we had the contours and the prescribed dose, we proceeded to import the position of the applicators and points, from the plan with C-arm images, to the study with sequential images, after which the upper edge of the tandem was matched with the hyperechogenic line representing the tandem in the ultrasound, in the three axes. As a result, as shown in Figure 4, we must have a 3D computer generated reconstruction of the applicator’s position in which the tandem should be in the thickness of the HR-CTV – aligned with the hyperechogenic line representing this—and the ring channels should not cross their paths with the HR-CTV or bladder. In addition, the bladder point should correspond anatomically to the balloon position of the Foley catheter in the ultrasound images.

### Comparison of the two plans and data collection

Plans were obtained for 20 applications (12 patients, 2 applications in 5 patients and 4 applications in 1 patient). Six patients had IIB clinical stage, one patient had IIIB, three patients had IIIC1, one patient had IIIC2 and one patient had IVA. Of the 20 applications, 14 were with tandem plus ring (4 with a 2.5-cm ring, 10 with a 3-cm ring and 6 with a 3.5-cm ring). The patients were asked to give their consent for the study, after which we proceeded to perform the insertion and planning with the C-arm images, taking the ultrasound images after and then removing the rectal probe to avoid the increase in dose by dispersion. The treatment was applied as indicated in ICRU 38, with the dose to prescription points and points of risk organs evaluated by the physician on duty; it was administered using a Cobalt-60 source. Then we proceeded to perform the steps already described for planning with ultrasound images, taking note of the results to perform descriptive and inferential statistics. Data were analysed using Python version 3.0.

The following variables were taken into account for collection: *point A dose* – dose to point A, as defined by ICRU 38, and *bladder dose* and *rectum dose*, which stand for their respective ICRU points. We also collected data of *HR-CTV*, *bladder volume* and* rectum volume*, as well as *D90HR-CTV* (dose delivered to 90% of HR-CTV), *D2cc bladder* (Maximum dose to 2cc of organ or target volume), *D1cc bladder, D2cc rectum, D1cc rectum* and the percentage of prescription dose (to point A) that was delivered to 90HR-CTV, which we named *ratio* for this purpose. All the volumes were outlined adapting the guidelines from GEC ESTRO to ultrasound image modality, as stated in in the *Processing and planning* section.

## Results

The means and SDs of the variables obtained in the data collection are shown in Table 1. The point A dose presents punctual variations in different cases, being the prescription dose; HR-CTV, bladder volume and rectum volume are according to anatomical knowledge and within the margins of previous studies that mention these values. It can also be seen that there are 6 cases where point A fell within the volume of the HR-CTV and there are 14 cases in which it did not. Ratio should be highlighted, evidencing an average of 104% with a great variability from 59.72% to 145.71%.

**Figure 5.**
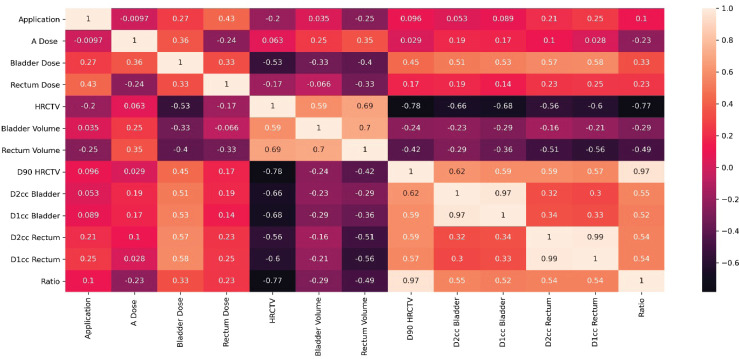
Map of the variables’ correlation.

**Figure 6.**
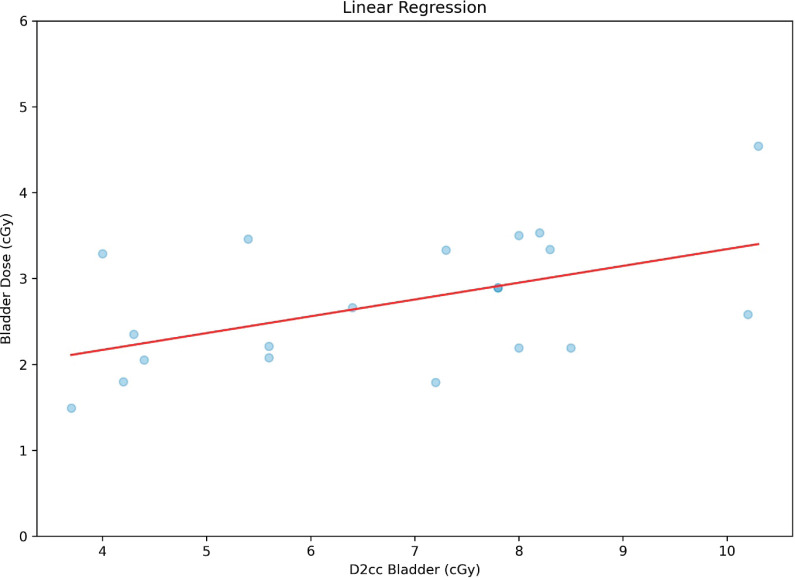
Correlation between bladder dose and D2cc bladder.

**Figure 7.**
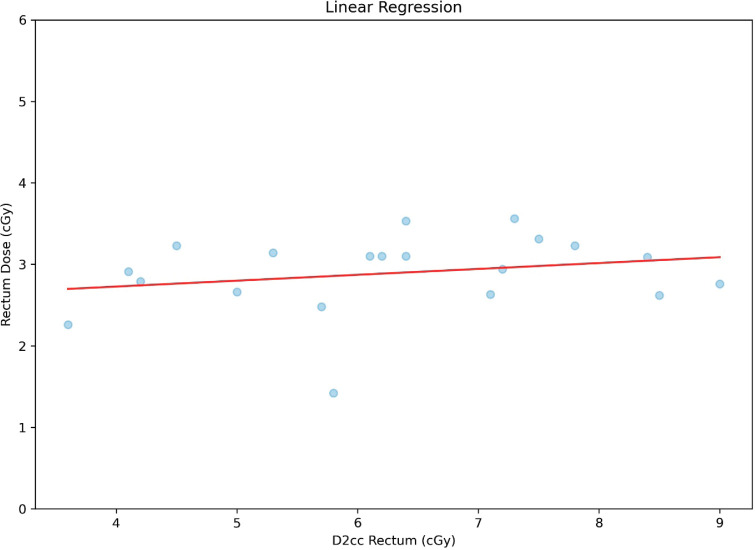
Correlation between rectum dose and D2cc rectum.

To have a bird’s-eye view of the correlation between the variables studied, a ‘heat map’ of the correlation was made with the Pearson correlation coefficient, giving it an overall look of the correlations, showing, among other interesting points, that there is a poor correlation between point A dose and D90HR-CTV. It is also noted that there is a poor and negative correlation between point A dose and ratio; this heat map is shown in Figure 5. A correlation analysis, correlation coefficient (*r*) and *p*-value (*p*), between bladder dose and D2cc bladder was performed, with a moderate and statistically significant correlation (*r* = 0.51 and *p* = 0.02), as shown in the graph in Figure 6. On the contrary, for the correlation analysis between rectum dose and D2cc rectum, there is no statistically significant correlation (*r* = 0.23 and *p* = 0.33), which can also be deduced by looking at Figure 7.

**Figure 8.**
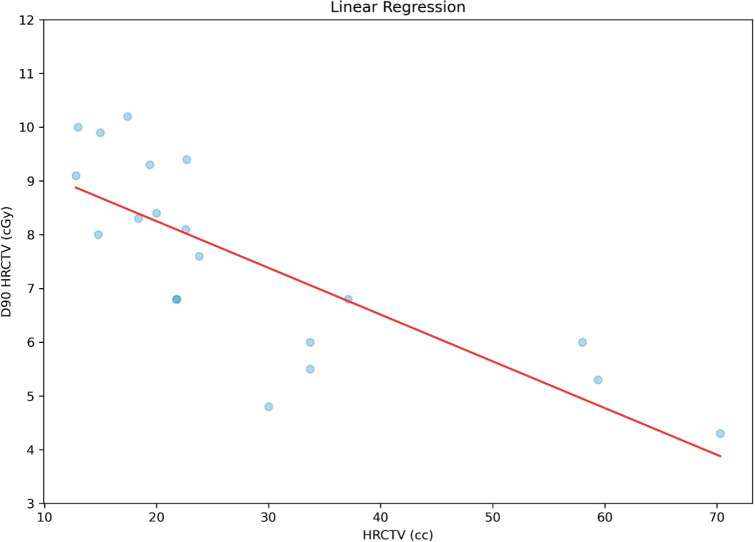
Correlation between D90HR-CTV and HR-CTV.

**Figure 9.**
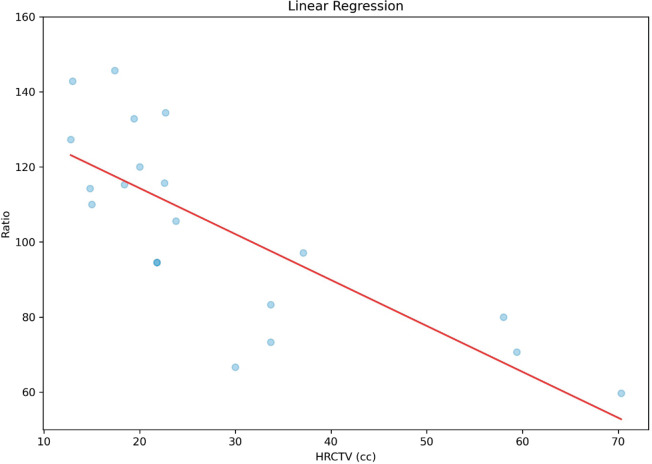
Correlation between ratio and HR-CTV.

Since the planning was carried out with orthogonal plates, prioritising the prescription to point A, which in all cases is adequate, we cannot have a correlation between point A dose and the D90HR-CTV as stated earlier. Correlation analysis was performed between HR-CTV and D90HR-CTV, which found a strong statistically significant inverse correlation (*r* = 0.78 and *p* < 0.0001), as shown in Figure 8. Similarly, a correlation was made between HR-CTV and ratio, which found a statistically significant inverse correlation (*r* = 0.77 and *p* < 0.0001), shown in Figure 9.

Finally, to determine the importance of the point A inclusion inside the HR-CTV, we proceeded to perform Student’s *t*-test analysis for independent samples, resulting in both variances being different with a statistical significance of *p* < 0.0001 (variance of point A inside HR-CTV = 0.707 and variance of point A outside HR-CTV = 2.2517, both have a normal distribution).

Student’s *t*-test analysis was performed for independent samples, stratifying D2cc bladder according to whether or not point A fell within the HR-CTV, which found a statistically significant difference in variances (*p* < 0.0001); in addition, the same analysis was performed for the D2cc rectum, which showed a statistical significance (*p* = 0.037).

## Discussion

The results obtained, adding image-guided brachytherapy in the context of cervical cancer treatment, are quite satisfactory, leading at 3 years, to a local control rate of 95%, a specific survival of 74% and an overall survival of 68% [6], which are better than those of its historical comparison. For proper realisation of these techniques, technology is required, which many centres do not have yet and probably will not have in the near future. In this sense, the use of alternatives, such as those offered by ultrasound, has been studied with good results. Van Dyk *et al* [7] reported a series of cases in which the first applicator insertion was done with MRI guidance, and subsequently, taking advantage of the imaging correlation between MRI and ultrasound, ultrasound images were obtained in the following plans to optimise treatment. In this study, local control at 3 years of 86%, overall survival of 75% and a cause-specific survival of 79% are reported. The use of transrectal ultrasound to guide treatment has also been described [8, 9], as well as the experimental use of an internal transducer, through the applicator, for proper tumour delineation and subsequent planning [10].

Variability has been found in several parameters, of which we must note that of the HR-CTV. This has already been described in other studies with much larger populations using tomography and MRI [6, 11] or ultrasound images [7] in which a wide range of sizes is shown. If we look for a possible explanation for this, in our daily practice, one of the most obvious factors is the fact that, as this is the only brachytherapy centre of the Ministry of Health in southern Peru, we receive patients from other hospitals that have already completed their treatment with external beam radiation therapy (EBRT), while our patients start their brachytherapy applications between receiving their external beam treatment. Thus, the effect of more of less EBRT fractions on tumour size is noted.

Adequate correlation has been found between bladder dose and D2cc bladder, while for the rectum, no such correlation has been found. This relationship has been approached differently in the available literature. In a 2008 study, adequate correlation between ICRU points and the dose at 2cc for rectum, but not for bladder, was found; however, correlation is understood as the similarity of both values [14]. A larger study, with 93 applications, concluded that the rectal ICRU point is a good substitute for the 2cc dose of rectum, but not the bladder point, with a large correlation of the ICRU dose with the 2cc dose in the first case [15]. In a 2018 study, it is found that the maximum dose to bladder is almost six times that of the ICRU point and almost five times in the case of rectum; however, both showed good correlation, indicating that both points underestimate the dose [16]. Studies further back in time reported that there is no good correlation between ICRU points and dose when using volumes [17–19].

The HR-CTV, understood according to the GEC ESTRO guidelines, presents important differences with the assumption of the A points as a prescription, in the sense that these points may fall within or out of the aforementioned volume, not corresponding to anatomical points suitable for prescription [12]. Some of the studies mentioned earlier show comparisons between the dosimetry of the A points with 2D technique and the A points placed using the volume [14, 15]. There is a great correlation between the two, with a difference of less than 12% in the three cases. The fact of having point A inside the HR-CTV, or not, is independent of the clinical stage; it seems to be dependent on the HR-CTV. The results of the Student *t*-tests for independent samples categorised by this variable (point A inside the HR-CTV or not) indicate that there is a difference between the variances of both D90HR-CTV and the ratio, bladder and rectum doses. The first two are explainable in themselves, and there are many publications which demonstrate the importance of volume for good conformation, indicating the use of interstitial needles in cases of large volumes [6, 11]. The difference of D2cc bladder and D2cc rectum, categorised according to whether the A points fall within the HR-CTV or not, is explained since in brachytherapy small distances give important results of dose variation, and the fact of having a large volume makes sure that, in addition, the risk organs are further away from the applicators.

Overall, we noted that there are statistically significant variations in the reported doses when we apply the plan designed with C-arm images and measure it with the volumes contoured in an ultrasound images-based plan. This difference points out the need, as we see, to further research this technique of acquisition, improving the acquisition technique and gear, and comparing the resulting volumes against other techniques that allow making volumes, such as CT or MRI.

## Conclusion

Implementation of this technique, although perfectible, could allow us to use the concept of volume in our clinical practice of brachytherapy. Ultrasound is a relatively inexpensive technique that could provide a volume structure for the realisation of better brachytherapy applications, being cost-effective. Analyses comparing hypothetical plans with ultrasound imaging sequence, versus 2D plans with C-arm images, show important differences. It warrants further research on the topic in order to assess its usefulness and its similarity to established volume techniques for brachytherapy, using CT or MRI.

## Abbreviations

HR-CTV: High-risk clinical target volume
ICRU: International Commission on Radiation Units and Measurements
D2cc: Maximum dose to 2cc of organ or target volume
CT: Computed tomography
MRI: Magnetic resonance imaging
TPS: Treatment planning system
D90HR-CTV: Dose to 90% of HR-CTV

## Conflicts of interest

The authors declare that they have no conflicts of interest.

## Funding

The authors state that this study was not funded.

## Figures and Tables

**Table 1.** Descriptive statistics of the variables.

Variable	*N*	Mean	SD	Minimal	Maximum
A dose	20	7.25	0.45	6.99	9.00
Bladder dose	20	2.71	0.77	1.49	4.54
Rectum dose	20	2.89	0.48	1.42	3.56
HR-CTV (cc)	20	28.28	16.41	12.80	70.30
Bladder vol. (cc)	20	311.55	146.76	151.80	631.00
Rectum vol. (cc)	20	35.06	17.45	10.80	65.60
D90HR-CTV	20	7.53	1.82	4.30	10.20
D2cc bladder	20	6.76	2.02	3.70	10.30
D1cc bladder	20	7.62	2.31	4.00	11.30
D2cc rectum	20	6.30	1.55	3.60	9.00
D1cc rectum	20	7.29	1.99	3.90	11.00
Ratio	20	104.20	26.01	59.72	145.71
